# Structural Analysis of Thymidylate Synthase from Kaposi’s Sarcoma-Associated Herpesvirus with the Anticancer Drug Raltitrexed

**DOI:** 10.1371/journal.pone.0168019

**Published:** 2016-12-09

**Authors:** Yong Mi Choi, Hyun Ku Yeo, Young Woo Park, Jae Young Lee

**Affiliations:** Department of Life Science, Dongguk University-Seoul, Ilsandong-gu, Goyang-si, Gyeonggi-do, Republic of Korea; Russian Academy of Medical Sciences, RUSSIAN FEDERATION

## Abstract

Kaposi’s sarcoma-associated herpesvirus (KSHV) is a highly infectious human herpesvirus that causes Kaposi’s sarcoma. KSHV encodes functional thymidylate synthase, which is a target for anticancer drugs such as raltitrexed or 5-fluorouracil. Thymidylate synthase catalyzes the conversion of 2′-deoxyuridine-5′-monophosphate (dUMP) to thymidine-5′-monophosphate (dTMP) using 5,10-methylenetetrahydrofolate (mTHF) as a co-substrate. The crystal structures of thymidylate synthase from KSHV (apo), complexes with dUMP (binary), and complexes with both dUMP and raltitrexed (ternary) were determined at 1.7 Å, 2.0 Å, and 2.4 Å, respectively. While the ternary complex structures of human thymidylate synthase and *E*. *coli* thymidylate synthase had a closed conformation, the ternary complex structure of KSHV thymidylate synthase was observed in an open conformation, similar to that of rat thymidylate synthase. The complex structures of KSHV thymidylate synthase did not have a covalent bond between the sulfhydryl group of Cys219 and C6 atom of dUMP, unlike the human thymidylate synthase. The catalytic Cys residue demonstrated a dual conformation in the apo structure, and its sulfhydryl group was oriented toward the C6 atom of dUMP with no covalent bond upon ligand binding in the complex structures. These structural data provide the potential use of antifolates such as raltitrexed as a viral induced anticancer drug and structural basis to design drugs for targeting the thymidylate synthase of KSHV.

## Introduction

Thymidylate synthase (TS) is highly conserved among all kingdoms of life including humans, mice, bacteria, protozoa, and some viruses. TS is an essential enzyme for DNA replication. It catalyzes the transfer of a methylene group from the cofactor 5,10-methylenetetrahydrofolate (mTHF) to its substrate 2′-deoxyuridine-5′-monophosphate (dUMP) and produces thymidine-5′-monophosphate (dTMP) and dihydrofolate (DHF) [1]. During methylation reaction, a nucleophilic reaction first occurs between the C6 atom of dUMP and sulfhydryl group of cysteine, creating a covalent bond. Subsequently, reductive methylation involves the transfer of a hydroxymethyl group from mTHF to dUMP [2,3]. Inhibition of TS disrupts DNA replication, leading to thymineless death in proliferating cells [4].

For several decades, many studies have revealed that human TS (hTS) could be an attractive target for cancer therapy, and drugs were developed to inhibit the enzymatic properties of hTS by mimicking its substrate or co-substrate [4]. Inhibitors targeting hTS, including 5′-fluorouracil, raltitrexed, and CB3717 (N10-propargyl-5,8-dideazafolic acid) are analogs of TS substrate or co-substrate; however, their clinical use is limited by drug resistance due to an increase in TS protein level. The binding of inhibitors to hTS can cause an increase in TS protein stability and up-regulation of protein expression [5,6]. This drug resistance has been attributed to the binding of inhibitors that restrict the auto-regulation of hTS translation [7]. According to a study on the autoregulation of TS translation, the ligand-free hTS protein inhibited the translation by binding to its mRNA and suppressing translation, while its ternary complex with dUMP and inhibitors impedes the binding of hTS to its mRNA [8]. hTS binds to its mRNA at two different regions in the autoregulation mechanism. It was reported that TS binding site 1 has a stem-loop structure including the translation initiation codon, and binding site 2 contains 200 nucleotides in the mRNA coding region [9,10]. Thus far, there are no structural reports of TS with its mRNA. Minimal sequences with an initiation start codon and immediate flanking sequences have been determined as an essential TS binding site [11]. In addition to enzymatic and translational autoregulation characteristics, hTS has been reported to down-regulate p53 translation by binding to the p53 mRNA coding region in some cell lines [12]. hTS induction is known to modulate p53 and p53-related gene expression in response to treatment with antifolate inhibitors [13].

Kaposi’s sarcoma-associated herpesvirus (KSHV) is an oncovirus that causes Kaposi’s sarcoma in AIDS patients, primary effusion lymphoma, and some types of multicentric Castleman’s diseases [14,15]. KSHV encodes functional thymidylate synthase (kTS), which shares 70% sequence identity with hTS [16]. The three-dimensional structures of TS have been reported for several different organisms including *E*. *coli* [17], *Lactobacillus casei* [18], *Bacillus subtilis* [19], rat [20], and human [21,22]. Recently, the crystal structure of varicella-zoster virus (VZV) TS was reported in apo and dUMP complex forms [23]. Owing to the limited information of viral TS and its ternary complex structure, structural analyses of KSHV TS and its complex structures with dUMP and raltitrexed (an inhibitor of hTS) were performed. The crystal structures of the apo, dUMP complex, and ternary complex structures were determined at 1.7 Å, 2.0 Å, and 2.5 Å, respectively. The overall structure of kTS was found to have a similar structural conformation to hTS, except minor differences in ligand binding site and conformational variations in some amino acids.

## Materials and Methods

### Expression and purification

The gene encoding kTS was cloned for four different constructs to optimize protein solubility and protein expression level. The four DNA constructs were ligated with the pET-28b (+) expression vector containing a 6X histidine at its N-terminus, digested by Nde1/Xho1 restriction enzymes (New England Biolabs, USA). BL21 Star (DE3) pLysS cells (Invitrogen, USA) were transformed with the recombinant plasmids. Transformed cells were grown in enriched Luria-Bertani medium at 310 K supplemented with 30 mg L^-1^ of kanamycin and chloramphenicol. When the cells reached an OD_600_ of 0.6, the proteins from the four constructs were induced with 1.0 mM isopropyl -D-1-thiogalactopyranoside (IPTG) and allowed to grow for another 4 h at 303 K. The cells were harvested by centrifugation at 2,300 *g* for 15 min at 277 K and immediately frozen at 203 K. The cell pellets (6~8 g) were thawed on ice and resuspended in buffer A (300 mM NaCl, 50 mM Tris-HCl pH 7.5, and 10% (*v*/*v)* glycerol) containing 1 mM phenylmethylsulfonyl fluoride (PMSF). Then, the cells were homogenized by ultrasonication (Vibra cell VCX750; Sonics, USA) at 277 K. Cell debris was removed by centrifugation at 36,000 *g* (Supra 22 K; Hanil BioMed Inc., Korea) for 1 h at 277 K. The supernatant was loaded onto a Ni-NTA column (GE Healthcare, UK) pre-equilibrated with buffer A, washed with buffer A containing 60 mM imidazole, and eluted from the column with buffer B (300 mM NaCl, 50 mM Tris-HCl pH 7.5, 10% glycerol (*v*/*v)*, and 300 mM imidazole). Among the four constructs of the kTS gene, the N-terminus truncated kTS (51–337 residues) alone was soluble whereas the other constructs (1–337, 51–324, and 1–324) were insoluble after expression. The N-terminus truncated kTS was further purified by size exclusion chromatography using a Superdex200 gel filtration column (GE Healthcare, UK), employing an elution buffer of 200 mM NaCl, 20 mM Tris-HCl (pH 8.0), 10%(*v*/*v)* glycerol, 1,4-dithiothreitol (DTT), and 1 mM EDTA. The purity of kTS was analyzed by 12% (*v/v*) SDS-PAGE. The purified protein was concentrated to 40 mg mL^-1^ using Centricon YM-10 (Millipore), and aliquots of the protein were stored at 193 K. The final purity was greater than 99% based on the appearance of a Coomassie blue-stained sample analyzed by SDS-PAGE.

### Crystallization

Crystallization of kTS was performed by sitting-drop vapor diffusion at 300 K. The protein solution containing 200 mM NaCl, 20 mM Tris-HCl (pH 8.0), 10% (*v/v*) glycerol, 1 mM DTT, and 1 mM EDTA was mixed with an equal volume of well solution containing 40 mM potassium phosphate (monobasic), 16% PEG 8K, 20% (*v/v*) glycerol in the drop and equilibrated with 80 μL of well solution in the well. Crystals appeared within 24 h and were grown as thin plate-shaped crystals for a week. The co-crystallization of kTS with dUMP and ralitrexed was unsuccessful. The complex crystals were obtained by soaking kTS crystals with dUMP or dUMP and raltitrexed. The well solution containing 6.3 mM dUMP and 5.6 mM raltitrexed was added to the drop containing the apo kTS protein crystals grown for 5 days, and incubation was carried out for 2 days at 300 K.

### Data collection and structure determination

The crystals were washed with the well solution, which is a suitable cryo-protectant solution. The directly mounted crystals were immediately flash-cooled in a stream of liquid nitrogen. X-ray diffraction was carried out using synchrotron radiation at 100 K with beamline SB-I at Pohang Accelerator Laboratory, Korea. The crystals were exposed to X-ray for 1 s per image, and frames were obtained in every 1° oscillation. The diffraction datasets were collected by ADSC Quantum 270 CCD image plate detector and processed using *DENZO* and *SCALEPACK* from the *HKL*-2000 program suite [24]. After apo kTS was solved by molecular replacement with *PHASER* from the *CCP4* program suite [25] using the structure of hTS [26] (PDB ID: IHVY, hereafter, hTS structure represents 1HVY) as an atomic coordinate, the complex structures of kTS were solved by molecular replacement using apo kTS as a searching model. All apo, binary, and ternary complex structures of kTS were refined with *REFMAC* from the *CCP4* program suite. Further manual building and refinement were carried out with *COOT* [27] and the *PHENIX* program package [28]. The refined model was finally evaluated using *MolProbity* [29]. The data collection and refinement statistics are presented in Table 1.The coordinates and structure factors have been deposited in the Protein Data Bank under accession numbers 5H38 for the apo kTS, 5H39 for the binary complex kTS, and 5H3A for the ternary complex kTS structure, respectively.

**Table 1:** Data collection and refinement statistics.

	Apo kTS	Binary complex (kTS+dUMP)	Ternary complex (kTS+dUMP+raltitrexed)
**Data collection**			
Resolutions range (Å)	50.00–1.70 (1.73–1.70)	50.00–2.00(2.03–2.00)	50.00–2.40(2.44–2.40)
Space group	P2_1_	P2_1_	P2_1_
Unit cell parameters (Å, °)	a = 61.52, b = 80.55, c = 65.60	a = 63.81, b = 78.86, c = 67.66	a = 63.41, b = 79.54, c = 67.28
	α = 90.0, β = 110.7, γ = 90.0	α = 90.0, β = 116.1, γ = 90.0	α = 90.0, β = 116.3, γ = 90.0
Number of observations	321,040	208,563	96,211
Unique reflections	65,947	40,983	23,609
Data completeness (%)	99.2 (99.6)	99.9 (100.0)	98.7 (98.5)
Redundancy	4.9 (4.9)	5.1 (5.1)	4.1 (4.1)
Average *I/σ(I)*	36.5 (4.8)	19.3 (2.6)	28.4 (2.7)
*R*_*merge*_^a^ (%)	6.7 (42.3)	7.3 (50.9)	5.9 (52.7)
**Refinement statistics**			
Resolution (Å)	20.0–1.70	20.0–2.00	20.0–2.40
No. of reflections	65,272	40,668	23,197
*R*_*work*_^b^ / *R*_*free*_ (%)	16.7 / 19.5	16.1 / 20.2	17.8 / 23.2
Rmsd bonds (Å)	0.007	0.008	0.009
Rmsd angles (°)	1.12	1.16	1.33
No. of protein residues	567	568	562
**Ramanchandran Plot** ^c^			
Most favored (%)	96.6	96.6	95.1
Allowed (%)	2.83	2.84	4.50
Outliers (%)	0.53	0.53	0.36
Rotamer outliers (%)	0.00	0.42	0.85

^a^ *R*_*merge*_ = ∑_h_∑_i_|*I*(*h*)_i_−<*I*(*h*)>|/∑_h_∑_i_*I*(*h*)_i_, where *I*(*h*) is the intensity of reflection *h*, ∑_h_ is the sum over all reflections, and ∑_i_ is the sum over i measurements of reflection *h*.

^b^ *R*_*work*_ = ∑ | |*F*_obs_|–|*F*_calc_| | / ∑ |*F*_obs_|, where *R*_free_ is calculated for a randomly chosen 5% of reflections, which were not used for structure refinement and *R*_work_ is calculated for the remaining reflections.

^c^ Determined using *Molprobity*

## Results

### Overall kTS structures

**Fig 1:**
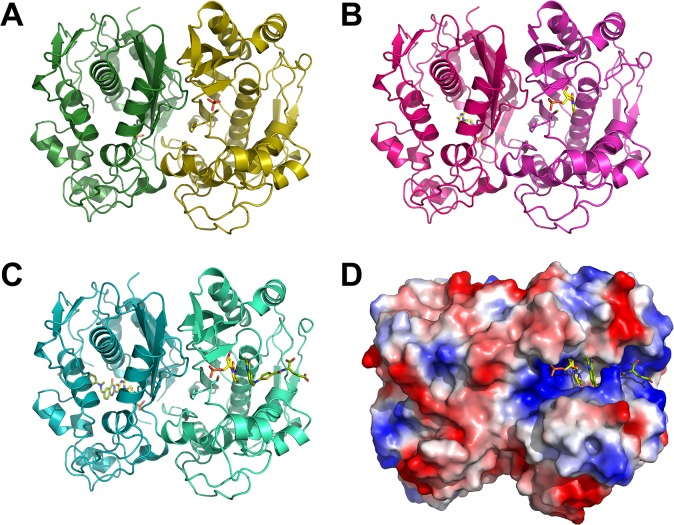
Overall kTS structures. (A), (B), and (C) represents the overall structures of apo, binary, and ternary kTS, respectively. kTS is a homodimer. Each monomer contains one phosphate in apo kTS, one dUMP in binary kTS, and one dUMP and one raltitrexed in ternary kTS. Each monomer is colored, with olive and forest in apo kTS, magenta and hot-pink in binary kTS, and green-cyan and teal in ternary kTS. Atoms N, H, O of the ligands are represented in blue, gray, and red, respectively. Atom C is colored in yellow in dUMP and chartreuse in raltitrexed. (D) Electrostatic potential of ternary kTS with dUMP and raltitrexed.

The crystals of kTS were obtained with the kTS proteins consisting of 51 to 337 amino acids, and the three different kTS structures, apo, binary complex, and ternary complex, were determined by molecular replacement at 1.7 Å, 2.0 Å, and 2.4 Å, respectively. The crystals of kTS contained a dimer per asymmetric unit with a solvent content of 43.17%, giving a crystal volume per protein mass of 2.16 Å^3^ Da^­1^ (calculated by *Matthews coef* of the *CCP4* program suite). The apo kTS structure (PDB code 5H38) was finally refined to R_work_ and R_free_ values of 16.7% and 19.5%, respectively, accounting for 567 amino acids of the dimer (Table 1). The complex structure of kTS with dUMP (PDB code 5H39) was refined to R_work_ and R_free_ values of 16.1% and 20.2%, respectively, accounting for 568 amino acids of the dimer including the dUMP molecule in each monomer. The ternary complex structure of kTS with dUMP and raltitrexed (PDB code 5H3A) was also refined to R_work_ and R_free_ values of 17.8% and 23.2%, respectively, accounting for 562 amino acids of the kTS dimer, and both dUMP and raltitrexed were positioned in each monomer (Fig 1). In apo kTS, phosphate molecules occupied the dUMP binding site. The loop region including amino acids Leu72 to Ile77 from one monomer and the last 2–6 amino acids in C-terminal region were disordered in kTS structures. kTS proteins were composed of 11 β-strands and 10 α-helixes and loops. The β-sheet containing Thr79–Phe83, Met85–Ser90, Leu221–Gln238, Ser240–Asp242, Glu271–Leu276, and Asp278–Tyr282 residues from each monomer was found to contact each other to form a dimer. Each monomer contained a ligand binding cleft in which the catalytic Cys219 residue was deposited.

The structures of kTS were almost identical except for some minor variations. The structures of kTS were well superimposed with root-mean-square deviation (r.m.s.d.) values of 0.37~0.41 Å (S1 Table). In the kTS homodimer, structural comparisons of each monomer revealed r.m.s.d values of between 0.25 Å and 0.39 Å. The solvent accessible surfaces (SASs) of apo, binary, and ternary kTS structures were calculated to 19,863 Å^2^, 19,604 Å^2^, and 19,877 Å^2^, respectively. These data clearly indicate that the binding of ligands did not have any influence on the structural conformation of kTS. In addition, a dual conformation was observed for the sulfhydryl group of catalytic Cys219, as shown in the electron density map of apo kTS.

### Structural comparison of kTS with other TS proteins

**Fig 2:**
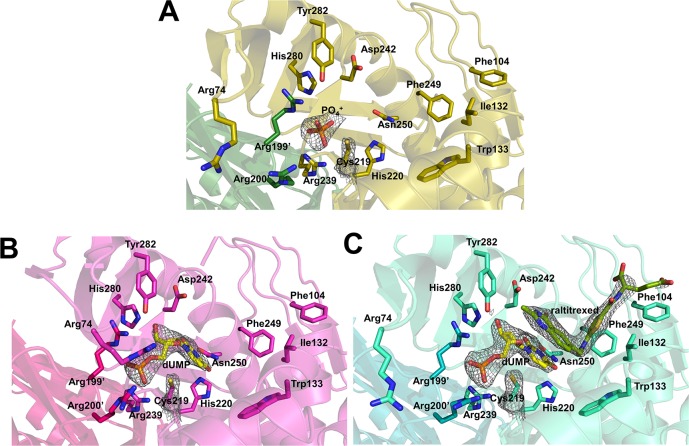
Ligand binding sites in kTS structures. (A), (B), and (C) represents apo (olive), binary complex (pink), and ternary complex kTS (cyan) structures, respectively. Atoms N, H, O of ligands are represented in blue, gray, and red, respectively. Atom C is colored in yellow in dUMP and chartreuse in raltitrexed. *2Fo-Fc* maps of catalytic Cys219, phosphate, dUMP, and raltitrexed are shown in apo, binary complex, and ternary complex kTS structures, respectively.

**Fig 3:**
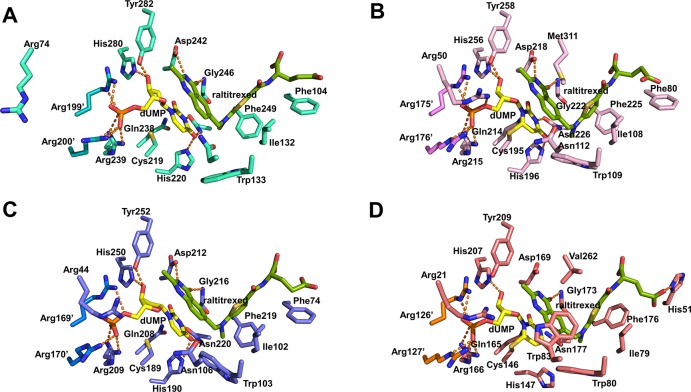
Structural comparison of the active site. (A), (B), (C), and (D) represents the active sites of kTS (cyan), hTS (light pink), rTS (slate), and ecTS (deep salmon), respectively. Atoms N, H, O of the ligands are represented in blue, gray, and red, respectively. Atom C is colored in yellow in dUMP and chartreuse in raltitrexed. Hydrogen bonds are represented by a dashed orange line.

Multiple sequence alignment and structural superimposition of TS proteins including kTS revealed that the overall structure of kTS shared high structural similarities with previously reported TS structures including human [21,22], rat [20], VZV [23] and *E*. *coli* [17] (S1 Fig and S1 Table). kTS was found to have over 70% sequence identity with rat TS (rTS), VZV TS (vTS), and hTS; however, it had 47% sequence identity with *E*. *coli* TS (ecTS). The superimposition of the kTS ternary complex on the other known ternary TS structures (hTS: 1HVY, rTS: 1RTS, and ecTS: 2KCE) revealed r.m.s.d. values of 0.9 Å, 0.8 Å, and 1.0 Å, respectively (S1 Table). The overall structural comparison between the binary complex of kTS and vTS proteins (4XSD and 4XSC) revealed high structural similarities with r.m.s.d. values of 0.76~0.84 Å. In addition, the rotation angles of subunit asymmetry (kappa in polar angles) between each subunit in TS structures were close to ~180°, indicated that TS proteins formed a dimer with almost two-fold symmetry regardless of ligand binding (S1 Table). TS proteins could adopt either open or closed conformations in their ternary complex structures. The ternary complex of kTS had an open conformation in which the catalytic Cys219 residue did not form a covalent bond with dUMP, which was also observed in rTS (Figs 2C and 3C). The ternary complexes of hTS and ecTS had closed conformations in which the catalytic Cys residue formed a covalent bond with dUMP. For kTS, the Arg74 residue in apo and ternary complex did not interact with in dUMP (Fig 2A and 2B), whereas the corresponding Arg residue in other TS structures was shown to interact with the phosphate group of dUMP (Fig 3). However, the loop region (Arg71–Thr79) containing Arg74 in the binary complex of kTS had a similar orientation to other TS structures.

### Ligand binding site

Although co-crystallization of kTS protein with ligands was unsuccessful, binary and ternary kTS structures were obtained by soaking apo kTS crystals with dUMP and/or raltitrexed. For the ternary kTS structure, an excellent density map of the ligands was observed except for the glutamate tail of raltitrexed (Fig 2C). The residues shown to interact with dUMP were His220, Arg239, Ser240, Asp242, Asn250, His280, Tyr282, Arg199’ (‘ indicates the other monomer), and Arg200’(Fig 2); the corresponding residues had the same interactions with dUMP in hTS (Fig 3). Three Arg residues (Arg199’, Arg200’, and Arg239) of binary and ternary kTS structures had direct contacts with the phosphate group of dUMP (Fig 2B and 2C), which were consistently observed in other TS structures (Fig 3). However, an additional arginine residue was shown to interact with the phosphate of dUMP in other TS structures, whereas the Arg74 residue in kTS was pointed out of the ligand binding site. The loop region (residues 71–79) of apo and ternary kTS structures was shifted away from the phosphate group, and the subsequent Arg74 residue was tilted out (Fig 2A and 2C); on the other hand, the Arg74 residue of binary kTS was coordinated around the phosphate group of dUMP as observed in other TS structures (Figs 2B and 3). Arg200’ residue in the apo kTS structure was rotated approximately 145° to the opposite direction of the phosphate group and was not observed to interact with dUMP. In addition to the difference in Arg coordination around the dUMP, slight differences in interaction with dUMP were observed. The hydrophobic interaction observed between the C2 atom of dUMP and Gln214 in hTS was absent in ternary kTS and rTS structures (Fig 3). A comparison between kTS and hTS revealed that the sulfhydryl group of catalytic Cys219 of kTS was rotated to approximately 20° away from dUMP. In addition, the sulfhydryl group of ternary kTS was 3.3 Å away from the C6 atom of dUMP.

The raltitrexed molecule was observed to bind to kTS at the active site by interactions with Phe104, Ile132, Asp242, Gly246, Phe249, Tyr282, and dUMP (Fig 2C). The overall binding of raltitrexed in kTS was similar to that in hTS and other TS proteins except for minor differences. There were less hydrophobic interactions with raltitrexed in kTS than in hTS. Glu87, Trp109, Asn112, and Met311 were involved in hydrophobic interactions with the raltitrexed molecule in the hTS structure, whereas the corresponding residues did not form hydrophobic interactions in ternary kTS and rTS structures. The Trp109 residue in hTS was found to interact with C7, C9, and CP1 of the raltitrexed molecule; the corresponding Trp133 residue in kTS did not interact with raltitrexed and was rotated about 60° away from raltitrexed (Fig 3A and 3B). A recent study on vTS structures indicated that the absence of the raltitrexed molecule may contribute to the open conformation of vTS. However, applying raltitrexed to kTS did not lead to the closed conformation as observed in rTS. In apo and ternary kTS structures, the C-terminal region (residues Glu334–Val337) was not assigned due to the lack of an electron density map. The C-terminal region was not involved in crystal lattice contacts. This region was considered to act as a lid of the ligand binding site by interacting with raltitrexed in the closed conformation in hTS and ecTS structures. In binary complex of kTS, this C-terminal region was well occupied around the ligand binding site, but it did not contribute to a closed conformation. The disordered C-terminal amino acids were consistently observed in the rTS structure, which had an open conformation. These observations suggest that the stable coordination of the C-terminal region of TS and co-substrate such as raltitrexed at the active site may be required to adopt a closed conformation.

## Discussion

The apo, binary complex, and ternary complex structures were determined by X-ray crystallography. TS protein is well conserved in many organisms of all kingdoms of life, and structural studies on it have been widely performed. Comparisons between kTS and other TS proteins revealed that kTS had high structural similarities and can be the target of hTS inhibitors. The overall structure of ternary kTS was similar to that of hTS, but the amino acid residues interacting with the ligands were slightly different. Superimposition indicated that dUMP and raltitrexed in kTS were positioned slightly outward from a ligand binding site compared to hTS (1HVY) whereas ligands were aligned well for hTS (1I00, open) and rTS (S2 Fig). Amino acids in contact with raltitrexed in kTS are less than in hTS. In addition, the amino acid residues lying in the disordered C-terminal region, which were considered to be in contact with raltitrexed in hTS, were not observed in kTS. The ternary kTS structure were determined by soaking apo kTS crystals with dUMP and raltitrexed since co-crystallization of kTS protein with ligands was unsuccessful. Although the C-terminal region did not make crystal contacts with other symmetry-related molecules, we could not exclude the possibility that ternary kTS structure was not able to adopt a closed conformation due to the crystal lattice which may impede conformational changes to a closed conformation. In addition, the resolutions of complex kTS crystal structures were deteriorated in accordance with ligand binding.

The distance between the Cα atoms of Tyr282 and Ile132 which were located at ligand binding site of kTS ternary complex was 18.8 Å, whereas the corresponding distance was 17.8 Å in hTS (S3 Fig). As calculated above, the distance across active site was over 18.5 Å in other TS structures with an open conformation, including rTS, binary kTS, and vTS structures; however, a shorter distance of 17.9 Å in ecTS demonstrated a closed conformation. C-terminal disorder might contribute to the open conformation in ternary kTS and rTS. Met311 at the C-terminus of hTS was revealed to participate in hydrophobic interactions with C8, C8A, N1 and C2 atoms of raltitrexed and push the raltitrexed molecule into the active site (Fig 3A and 3B). The C-terminal region was observed in the binary kTS structure, but with an open conformation. These results suggest that the absence of hydrophobic interactions with the C-terminal region and raltitrexed may lead to the open conformation but the hydrophobic interactions with the C-terminal region and raltitrexed may be required to adopt a closed conformation in the TS structures.

Raltitrexed has been used as an antimetabolite drug targeting TS in cancer chemotherapy [30,31]. Raltitrexed treatment in cancer cell lines has shown to induce the stabilization and over-expression of hTS protein [5,6]. These structural studies on kTS bound with dUMP and raltirexed, as well as ligand-free and dUMP complex forms provide further insight into the antiviral drug development. Additional studies pertaining to the selectivity of viral TS proteins are necessary to overcome the limitation of antifolate as an antiviral drug.

## Supporting Information

S1 Fig Multiple sequence alignment of TS proteins. Highly conserved residues and partially conserved residues are shaded in green and orange, respectively. The conserved residues at the active site are indicated by the red triangles.
(TIF)

S2 Fig Superimposition of the active site in TS structures. (A), (B), (C), and (D) represent structural alignments of kTS (5H3A, open) to hTS (1I00, open), rTS (1RTS, open), hTS (1HVY, closed), and ecTS (2KCE, closed), respectively. Each structure is colored in cyan, light blue, slate, light pink, and deep salmon, respectively. Atoms N, H, O of the ligands are represented in blue, gray, and red, respectively. Amino acids are drawn faintly except catalytic cysteine residue and ligands.
(TIF)

S3 Fig Comparison of the active site in TS structures. (A), (B), (C), and (D) represent distances across the active site of ternary kTS (cyan), hTS (light pink), rTS (slate), and ecTS (deep salmon), respectively. (E) and (F) represent distances across the active site of binary kTS (hot pink) and binary vTS (wheat), respectively. Atoms N, H, O of the ligands are represented in blue, gray, and red, respectively. Atom C is colored in yellow in dUMP and chartreuse in raltitrexed. Distances are represented by a dashed black line.
(TIF)

S1 File Apo kTS coordinate. (CIF)

S2 File Apo kTS structure factor. (CIF)

S3 File Validation report of apo kTS structure. (PDF)

S4 File Binary kTS coordinate. (CIF)

S5 File Binary kTS structure factor. (CIF)

S6 File Validation report of binary kTS structure. (PDF)

S7 File Ternary kTS coordinate. (CIF)

S8 File Ternary kTS structure factor. (CIF)

S9 File Validation report of ternary kTS structure. (PDF)

S1 Table R.m.s.d between kTS structures and other TS structures. (DOCX)
