# Supplementary figures and images for: Structural Analysis of Thymidylate Synthase from Kaposi’s Sarcoma-Associated Herpesvirus with the Anticancer Drug Raltitrexed
Source: PLoS One. 2016 Dec 9;11(12):e0168019. doi: 10.1371/journal.pone.0168019 (PMC5148040; doi:10.1371/journal.pone.0168019)

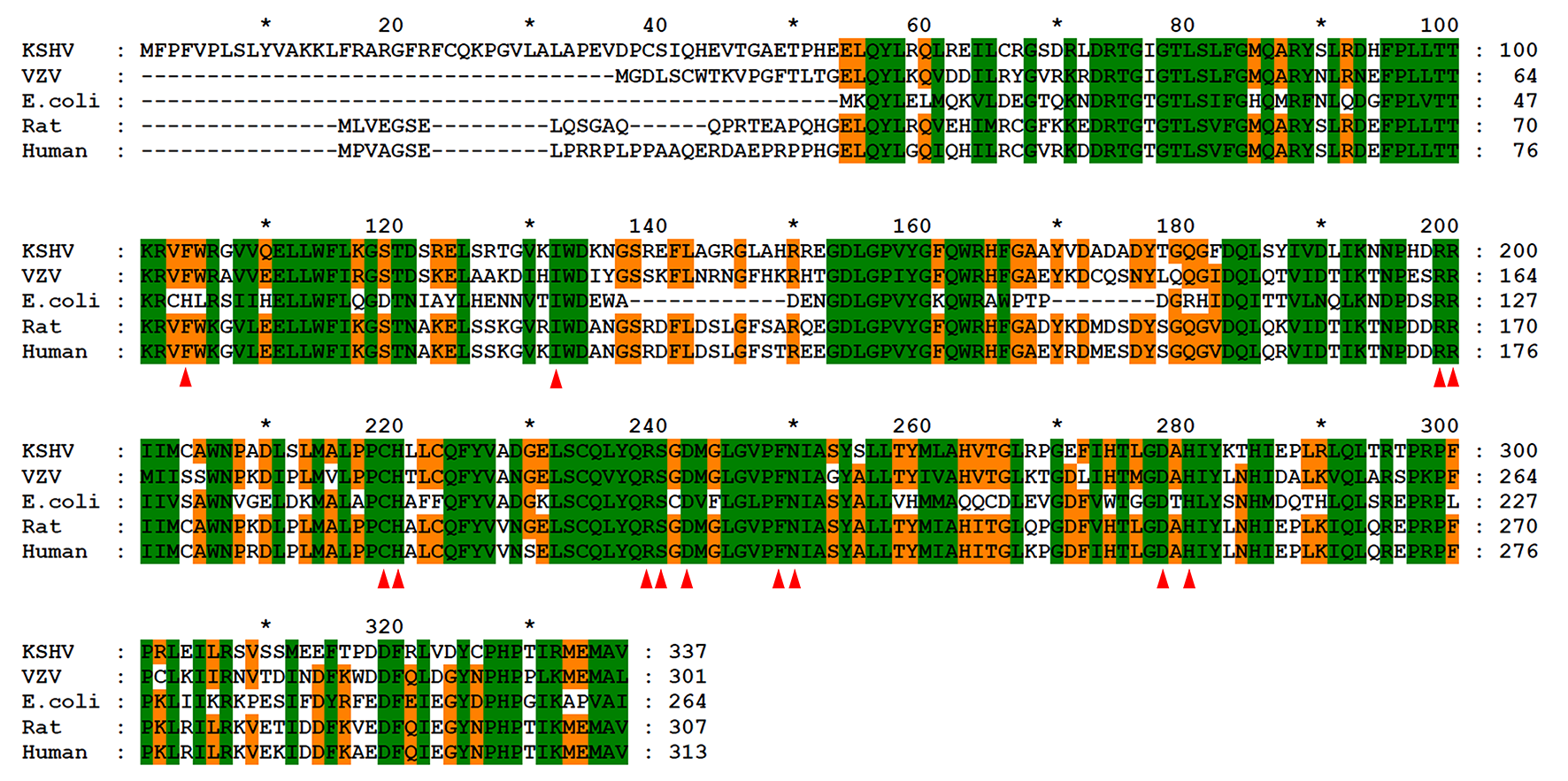

Supplement: S1 Fig — Highly conserved residues and partially conserved residues are shaded in green and orange, respectively. The conserved residues at the active site are indicated by the red triangles. (TIF) [file pone.0168019.s001.tif]

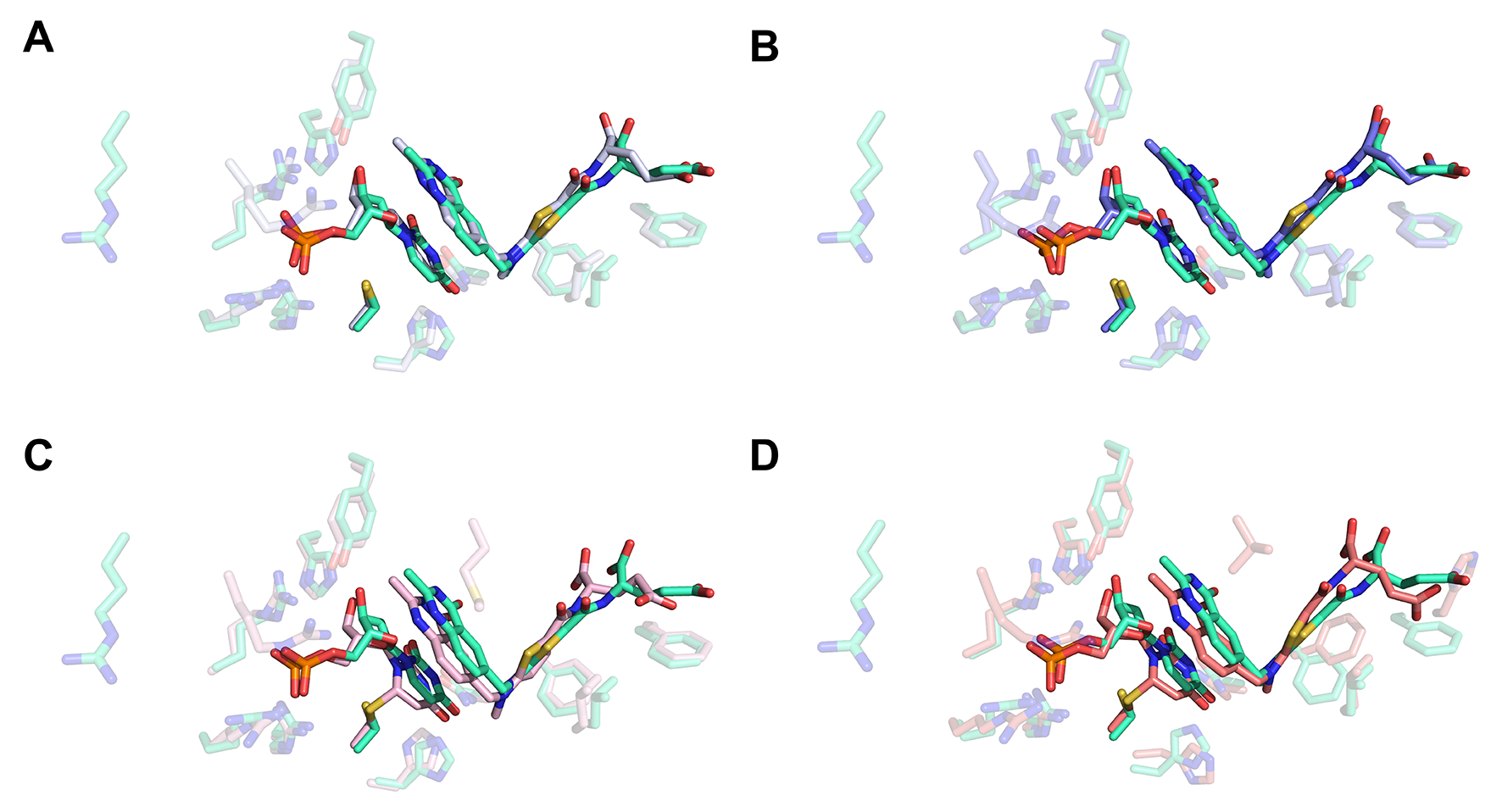

Supplement: S2 Fig — (A), (B), (C), and (D) represent structural alignments of kTS (5H3A, open) to hTS (1I00, open), rTS (1RTS, open), hTS (1HVY, closed), and ecTS (2KCE, closed), respectively. Each structure is colored in cyan, light blue, slate, light pink, and deep salmon, respectively. Atoms N, H, O of the ligands are represented in blue, gray, and red, respectively. Amino acids are drawn faintly except catalytic cysteine residue and ligands. (TIF) [file pone.0168019.s002.tif]

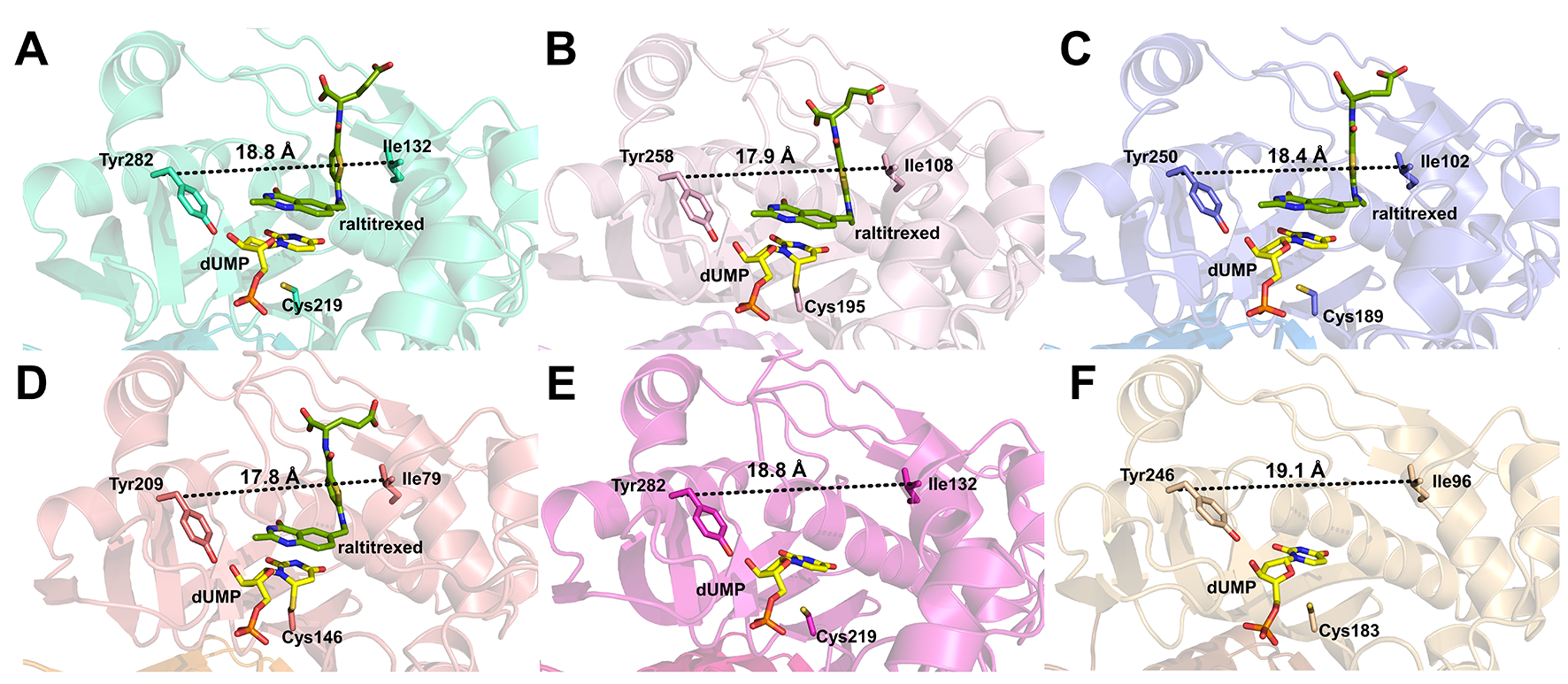

Supplement: S3 Fig — (A), (B), (C), and (D) represent distances across the active site of ternary kTS (cyan), hTS (light pink), rTS (slate), and ecTS (deep salmon), respectively. (E) and (F) represent distances across the active site of binary kTS (hot pink) and binary vTS (wheat), respectively. Atoms N, H, O of the ligands are represented in blue, gray, and red, respectively. Atom C is colored in yellow in dUMP and chartreuse in raltitrexed. Distances are represented by a dashed black line. (TIF) [file pone.0168019.s003.tif]
