# Supplementary material for: Structural Analysis of Thymidylate Synthase from Kaposi’s Sarcoma-Associated Herpesvirus with the Anticancer Drug Raltitrexed
Source: PLoS One. 2016 Dec 9;11(12):e0168019. doi: 10.1371/journal.pone.0168019 (PMC5148040; doi:10.1371/journal.pone.0168019)
Supplement: S1 Table — (DOCX) [file pone.0168019.s013.docx]

**S1 Table. R.m.s.d between kTS structures and other TS structures**

|  | **Each monomer (Å, °)** ^a^ | **Apo kTS (Å)** | **Binary kTS (Å)** | **Ternary kTS (Å)** |
| --- | --- | --- | --- | --- |
| **Apo kTS (5H38)** | 0.25 / 179.66 |  | 0.41 | 0.37 |
| **Binary kTS (5H39, open)** | 0.39 / 179.84 |  |  | 0.40 |
| **Ternary kTS (5H3A, open)** | 0.35 / 179.98 |  |  |  |
| **Apo hTS (1HW4, open)** | - / 180.0 | 1.91 | 1.84 | 1.83 |
| **Ternary hTS (1I00, open)** | 0.41 / 179.78 | 1.09 | 0.99 | 1.01 |
| **Ternary hTS (1HVY, closed)** | 0.28 / 179.41 | 0.88 | 0.87 | 0.89 |
| **Ternary rTS (1RTS, open)** | 0.18 / 179.83 | 0.95 | 0.94 | 0.93 |
| **Ternary ecTS (2KCE, closed)** | 0.33 / 179.26 | 1.00 | 1.00 | 0.99 |
| **Binary vTS (4XSD, open)** | 0.66 / 179.53 | 0.89 | 0.84 | 0.87 |

^a^ Rotation angle (κ) of each monomer from polar angles (φ,ψ,κ ) were calculated by *Lsqkab* in *CCP4* program suit.
